# Fatty Liver as a Potential Surrogate for Waist Circumference in the Diagnosis of Metabolic Syndrome: A Population-Based Study among Chinese Adults

**DOI:** 10.1155/2018/7903982

**Published:** 2018-04-10

**Authors:** Boren Jiang, Ting Gu, Kun Zhou, Yanjun Zheng, Yuyu Guo, Yingli Lu

**Affiliations:** ^1^Department of Endocrinology and Metabolism, Shanghai Ninth People's Hospital, Shanghai Jiao Tong University School of Medicine, Shanghai, China; ^2^Department of Gastroenterology, Shanghai Ninth People's Hospital, Shanghai Jiao Tong University School of Medicine, Shanghai, China

## Abstract

Increased waist circumference (WC) is an essential component of metabolic syndrome (MetS). Asians have been found to have more intra-abdominal adipose tissue than Caucasians, in spite of having smaller WC. The purpose of this study was to explore whether NAFLD could be used as a surrogate for MetS diagnosis in the normal WC population. A total of 9694 Chinese residents were selected from SPECT-China, a population-based survey of Chinese adults aged ≥ 18 years in East China. Insulin resistance was calculated by the homeostatic model assessment for insulin resistance (HOMA-IR). MetS z-score was used to evaluate the degree of total metabolic disorder. Logistic regression models were used to obtain the associations between different categories and metabolic syndrome (MetS) components. The prevalence of NAFLD was 27.6%, in which 35.2% were without abnormal WC. Subjects with only NAFLD had similarly severe insulin resistance and OR for clustering of MetS components compared with those with only abnormal WC. A considerable number of NAFLD cases, although had severe metabolic disorder, would not be detected by the current MetS definition. NAFLD could be used as a potential surrogate marker for the diagnosis of MetS in normal WC population.

## 1. Introduction

The term metabolic syndrome (MetS), also known as insulin resistance syndrome, refers to a cluster of metabolic abnormalities that are either causes or consequences of insulin resistance and that directly increase the risk of cardiovascular atherosclerotic diseases (CVD) and diabetes mellitus type 2 (T2DM) [1]. Since the first formalized definition of MetS was introduced in 1998, at least six different sets of criteria have been proposed for MetS. The most recent version defines MetS as a disorder that includes any three of the following five: increased waist circumference (WC), increased fasting plasma glucose or type 2 diabetes, hypertriglyceridaemia, low HDL cholesterol, or hypertension [2]. Among the five, increased WC is seen as an essential component. The reason is that central obesity, as assessed by WC, is highly correlated with insulin sensitivity, cardiovascular disease, and the other metabolic syndrome components, and central obesity could be an early step in the etiological cascade leading to full metabolic syndrome [3].

Compared with white Caucasians, Asians have been found to have a high percentage of body fat and a significantly increased risk for cardiovascular disease and diabetes even among those with a normal body mass index (BMI) [4]. At a given percentage of body fat, BMI values of Asians were 3 kg/m^2^ lower than those in white Caucasians. Similarly, Asians have been found to have more intra-abdominal adipose tissue than Caucasians, in spite of having smaller waists [5]. The current MetS diagnostic criteria would lead to many of the metabolically unhealthy subjects without higher WC not identified.

NAFLD is the hepatic manifestation of MetS and frequently coexists with obesity and insulin resistance. The amount of fat in the liver correlates closely with the amount of visceral fat and is a good indicator of insulin resistance [6]. In recent years, ultrasonography was used as a routine screening tool for NAFLD and more and more NAFLD without higher WC were identified. The purpose of this study was to explore whether NAFLD, another accurate indicator for visceral obesity and insulin resistance, could be used as a surrogate for MetS diagnosis in the normal WC population.

## 2. Subjects and Methods

SPECT-China (ChiCTR-ECS-14005052) is a population-based cross-sectional survey on prevalence of metabolic diseases and risk factors in East China. Recruitment and enrollment have been described in detail [7]. Chinese citizens ≥ 18 years old who had lived in their current area for ≥6 months were selected. We excluded subjects with severe communication problems, acute illness, or who were unwilling to participate. From February 2014 to December 2015, a total of 10441 subjects were finally recruited form twenty-two residential sites in Shanghai, Zhejiang, Jiangxi, Jiangsu, and Anhui Provinces. The study was approved by the Ethics Committee of Shanghai Ninth People's Hospital, Shanghai Jiao Tong University School of Medicine. Informed consent was obtained from all patients included in the study. For participants who were illiterate, we obtained written informed consent from their proxies.

Individuals were excluded who had missing WC (*n* = 388) or abdominal ultrasonographic results (*n* = 390), had pure alcohol consumption > 40 g/day for men or >20 g/day for women (*n* = 11), had active viral hepatitis (*n* = 17), history of cirrhosis or hepatic carcinoma (*n* = 9) or liver cirrhosis signs upon abdominal ultrasonography (*n* = 18), and had long-term (>3 months) medications related to NAFLD (corticosteroids, estrogen, amiodarone, and methotrexate) (*n* = 22). Ultimately, a total of 9694 subjects (females, 5702) were included in our data analysis.

## 3. Measurements

Subjects were interviewed face-to-face to complete pretested questionnaires covering sociodemographic information, medical history of coronary heart diseases, diabetes, hypertension, and lipid disorders. WC was measured on standing participants midway between the lower edge of the costal arch and the upper edge of the iliac crest using a nonelastic tape (to the nearest 0.5 cm). The measurements were taken in duplicate, and the averages of these measurements were used in the analyses. Resting systolic and diastolic BP was measured three times at 1 min intervals using a standard mercury sphygmomanometer after a 5-minute rest. The average of the second and the third readings was used in the analyses [8].

Fasting blood samples were collected from all participants after an 8-hour overnight fast. Serum high-density lipoprotein cholesterol (HDL-C), triglyceride (TG), and fasting blood glucose (FBG) were analyzed enzymatically using a Beckman Coulter AU 680 (Beckman Coulter) instrument. Insulin resistance was estimated by calculating the homeostatic model assessment for insulin resistance (HOMA-IR) index [9], and the fasting insulin (FINS, pmol/L) × fasting glucose (mg/dL)/(22.5 × 6.965) levels were measured.

Three trained ultrasonographers performed abdominal ultrasound examinations using an ultrasound device (Mindray M7, Shenzhen, China) and were blinded to the participants' history of liver disease, biochemical data, or clinical findings. Of 4 known criteria (hepatorenal echo contrast, liver brightness, deep attenuation, and vascular blurring), the participants were required to have hepatorenal contrast and liver brightness to be given a diagnosis of NAFLD [10, 11].

## 4. Definition of Metabolic Disorders

MetS was defined based on the IDF/AHA harmonized criteria [2], including WC ≥ 90 cm in men or ≥80 cm in women [4, 12], HDL cholesterol < 1.0 mmol/L in men or <1.3 mmol/L in women, TGs ≥ 1.7 mmol/L or taking lipid-lowering drugs, FBG level ≥ 5.6 mmol/L or taking hypoglycemic drugs, and BP ≥ 130/85 mmHg or taking antihypertensive drugs.

For each risk factor, a z-score was calculated (individual value − sample mean/standard deviation of the sample). For blood pressure, the MAP (2/3 DBP + 1/3 SBP) was used for calculating the score. In the present study, participants were grouped by WC; thus, we calculated the MetS z-score (four factors) = BP z-score + glucose z-score + HDL-C z-score + triglycerides z-score. It takes into account continuous changes in each component, representing the score of continuous risk for MetS [13]. A lower risk score is indicative of a better metabolic profile.

## 5. Statistical Analysis

Data analyses were performed using IBM SPSS Statistics, version 22 (IBM Corporation, Armonk, NY, USA). All analyses were two-sided. A *P* value < 0.05 indicated significance. Continuous variables were expressed as median (interquartile range) and categorical variables as a percentage (%). To test for differences of metabolic parameters among the four groups, generalized linear model was used after adjusting for age and sex. FBG was normalized by inverse transformation. HOMA-IR, insulin concentration, and TG were normalized by logarithmic transformation. The estimates of the associations between WC, fatty liver, and MetS components were analyzed using multiple logistic regressions. Estimates were then adjusted for age, sex, living area, occupation, and smoking status.

## 6. Results

The participants were classified into 4 study groups according to their WC and ultrasound findings. Group 1 (normal WC without NAFLD) included those without NAFLD or higher WC (WC < 90 cm for males and WC < 80 cm for females). Group 2 (normal WC with NAFLD) included those without a higher WC but who had FL. Group 3 (abnormal WC without NAFLD) included those with a higher WC but no FL, and Group 4 (abnormal WC with NAFLD) included those with both a higher WC and FL.

The basic characteristics of the study population were shown in Table 1. Of 9694 individuals, 2671 (27.6%) had NAFLD, in which 941 (35.2%) were without higher WC. Subjects in group 2 (normal WC with NAFLD) were younger than those in group 3 (abnormal WC without NAFLD). There was no difference in the ages of normal WC subjects without or with NAFLD (groups 1 and 2). Compared with group 1 or 3, subjects in group 2 had more urban residents, less manual worker, and more current tobacco smokers. As to the median values for the components of MetS, subjects in group 2 had higher TG, BP, FBG, basal insulin, HOMA-IR, and MetS z-score and lower HDL-C levels than in group 1. Compared with group 3, group 2 members had higher TG and MetS z-score, but lower SBP levels. There were no significant differences in the values of HDL, FBG, basal insulin, and HOMA-IR between groups 2 and 3. Because medication could affect the metabolic variables, we repeated the statistical analysis after excluding patients who were taking medications for diabetes, hypertension, or hyperlipidemia and obtained similar results (Supplementary Table 1).

**Figure 1:**
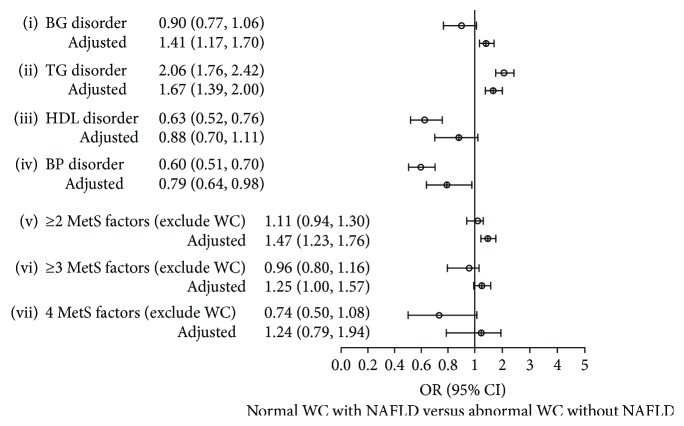
Adjusted ORs and 95% CI for MetS components among group 2 (normal WC with NAFLD) and group 3 (abnormal WC without NAFLD). Group 3 was used as reference group. Data were adjusted for age, sex, living area, occupation, and smoking status. CIs that do not cross the line (1.0) were considered statistically significant. ≥*n* MetS factors: having any kind of combinations of ≥*n* disorders (BG, TG, HDL, and BP).

The frequency of MetS component for each WC/NAFLD group and the odds ratios (ORs) for developing an abnormality among the MetS components are shown in Table 2. Using group 1 (normal WC without NAFLD) as reference, subjects in groups 2 (normal WC with NAFLD) and 3 (abnormal WC without NAFLD) had greater ORs for developing four MetS component abnormality and for having 2 or more MetS components. Compared with group 3 (Figure 1), group 2 subjects had a higher OR for TG disorder (OR = 1.67; 95% CI = 1.39–2.00; *P* < 0.01) and BG disorder (OR = 1.41; 95% CI = 1.17–1.70; *P* < 0.01), but a lower OR for BP disorder (OR = 0.79; 95% CI = 0.64–0.98; *P* < 0.05). As to the combination of disorders (except WC), group 2 members had a higher OR (OR = 1.47; 95% CI = 1.23–1.76; *P* < 0.01) for having ≥ 2 MetS components and marginally higher OR (OR = 1.25; 95% CI = 1.00–1.57; *P* = 0.054) for having ≥ 3 MetS components than group 3, but the ORs showed no differences for having 4 components.

The values of insulin resistance and MetS z-score of 2, 3, or 4 disorder combinations for each WC/NAFLD group were shown in Table 3. Among the subjects with 2 disorders, group 3 (abnormal WC without NAFLD), which could be diagnosed as MetS, had similar insulin concentration, HOMA-IR, and MetS z-score with group 2 (normal WC with NAFLD). However, group 2 members with 2 disorders could not be classified as MetS, despite increased basal insulin, HOMA-IR, and MetS z-score. In subjects with 3 or 4 disorders, the values of HOMA-IR and MetS z-score showed no differences between group 2 and group 1 or group 3.

## 7. Discussions

In the present study, we found that subjects with only NAFLD (i.e., no abnormal WC) had similarly severe insulin resistance and roughly equal OR for clustering of MetS components compared with those with only abnormal WC (i.e., no NAFLD). The NAFLD subjects plus two MetS components had similarly severe insulin resistance and metabolic disorder with the abnormal WC subjects plus two MetS components. Our data suggest that NAFLD could be used as a surrogate marker for the diagnosis of MetS in the normal WC population.

In our population, 35.2% NAFLD subjects were with normal WC. A strong correlation has been proved between NAFLD and abdominal obesity [14]; few researches have involved the prevalence of subjects with only NAFLD or only abnormal WC. These subgroups were particularly important for Asians. The prevalence of NAFLD in the East (15–30% in China and 20–30% in Japan, resp.) was probably as common if not more than in the West. Patients with NAFLD in the East had a lower BMI than patients in the West, as well as lower rates of overweight and obesity [15]. Data from Japan indicated that only 15–20% of affected patients are obese [16]. A large group of people in Asia had only NAFLD (i.e., no abnormal WC). Learning more characteristics about these subgroups of population is useful for clinical practice.

In the five components of MetS, the anthropometric component WC was regarded as an indicator for visceral obesity and insulin resistance. Emerging evidence suggests that, compared with visceral adipose tissue, liver fat is another important determinant of the metabolic complications of obesity [17–19]. Theoretically, NAFLD, as another adipose tissue compartments, can be used as a surrogate for visceral obesity. Several studies even demonstrated that the quantity of liver fat was more closely linked to the metabolic complications of obesity than that of visceral fat. Chang and Chen displayed subjects with only NAFLD had a greater odds ratio for developing abnormal MetS components than those with only abnormal WC [20]. Stefan et al. reported that liver fat had stronger correlation with insulin sensitivity than visceral adipose tissue among the obese subjects [19]. These and most other NAFLD studies have been limited to smaller samples selected for metabolic and obesity-related traits. In our population-based study, compared with those with only abnormal WC, subjects with only NAFLD had similarly severe insulin resistance and roughly equal OR for clustering of MetS components. These meant that NAFLD could be a surrogate for abnormal WC in detecting subjects with insulin resistance and metabolic disorders among Chinese population.

Taking the number of MetS components in consideration, those individuals who demonstrated two components could be diagnosed as MetS in only abnormal WC group (group 3), but not in only NAFLD group (group 2). However, group 2 had similar insulin levels, HOMA-IR, and MetS z-score with group 3, which suggest that a considerable number of NAFLD cases, although had severe metabolic disorder, would not be detected by the current diagnostic criterion.

Of note, despite similar insulin resistance and total metabolic disorders, the extent of association with individual risk factor differed between subjects with only NAFLD and only abnormal WC. Specifically, NAFLD had a higher likelihood for BG and TG disorder, while abnormal WC appeared to be associated with more BP disorder. The liver is the main regulator of glucose and lipid in the body. In a report from the Framingham Heart Study, fatty liver was significantly associated with dyslipidemia and dysglycemia independently of visceral adipose tissue (VAT) [21]. Liver fat also appears to be a better marker than VAT in determining categories of prediabetes, with an increasing trend in liver fat across groups characterized by normal glucose tolerance, isolated impaired fasting glucose (IFG), impaired glucose tolerance (IGT), and combined IFG and IGT [17]. Lots of studies have proven the association between visceral obesity and hypertension, but the liver is known to play little known role in blood pressure regulation [22]. The exact mechanisms of MetS component difference between abnormal WC and NAFLD are unclear yet, which may be related to the metabolites and cytokines released from the two depots [23–25]. Future prospective studies are also warranted to study their difference on the development of CVD and T2DM.

The present study has some limitations. First, the sensitivity and specificity of ultrasonography for detecting NAFLD are a constant matter of debate [26]. Second, the cross-sectional nature of the study did not allow definitive conclusions about causal relationships to be drawn between NAFLD and abnormal WC with insulin resistance and metabolic disorders. Third, as mentioned above, the prevalence and characteristics of NAFLD and visceral obesity vary in different races or geographical locations. Our study was limited by including only individuals of Chinese ancestry and thus cannot be generalized to other ethnicities.

## 8. Conclusions

Based on our population-based study, compared with those with only abnormal WC, subjects with only NAFLD had similarly severe insulin resistance and roughly equal OR for clustering of MetS components. A considerable number of NAFLD cases, although had severe metabolic disorder, would not be detected by the current MetS diagnostic criterion. NAFLD could be used as a potential surrogate marker for the diagnosis of MetS in normal WC population.

## Figures and Tables

**Table 1:** Basic characteristics of the study population, grouped by WC and NAFLD.

	Normal WC		Abnormal WC	
	Without NAFLD (1)	With NAFLD (2)	Without NAFLD (3)	With NAFLD (4)
	*n* = 5159	*n* = 941	*n* = 1864	*n* = 1730
Sex (women), *n* (%)	3014 (58.4)	273 (29.0)^ab^	1358 (72.9)	1057 (61.1)
Age, yrs	51 (41, 61)	52 (42, 60)^b^	60 (51, 68)	57 (49, 64)
Components of MetS				
WC, cm	74 (69, 78)	81 (77, 86)^ab^	88 (83, 93)	92 (86, 97)
TG, mmol/L	1.1 (0.83, 1.51)	1.73 (1.2, 2.46)^ab^	1.38 (1.02, 1.94)	1.89 (1.4, 2.67)
HDL, mmol/L	1.47 (1.27, 1.69)	1.3 (1.11, 1.49)^a^	1.38 (1.18, 1.59)	1.28 (1.12, 1.47)
SBP, mmHg	124 (112, 139)	130 (118, 146)^ab^	137 (123, 152)	140 (126, 155)
DBP, mmHg	76 (68, 84)	81 (73, 89)^a^	81 (73, 89)	85 (77, 93)
FPG, mmol/L	5.2 (4.86, 5.63)	5.34 (4.92, 5.95)^a^	5.37 (4.94, 5.96)	5.59 (5.10, 6.46)
Insulin resistance				
Insulin, pmol/L	28 (20.2, 38.8)	35.75 (25.3, 50.95)^a^	36.75 (26.5, 51)	48 (35, 67.6)
HOMA-IR	0.95 (0.67, 1.32)	1.29 (0.86, 1.79)^a^	1.29 (0.91, 1.82)	1.83 (1.25, 2.65)
MetS z-score (4 factors)	−2.44 (−3.67, −1.03)	−0.80 (−2.06, 0.83)^ab^	−0.88 (−2.27, 0.73)	0.48 (−0.99, 2.39)
Measures of liver function				
AST, U/L	22 (19, 27)	24 (20, 30)^a^	23 (20, 28)	25 (21, 31)
ALT, U/L	16 (12, 22)	22 (16, 32)^ab^	17 (13, 23)	23 (17, 34)
Living area, %				
Rural	58.50	41.10^ab^	63.40	59.10
Urban	41.50	58.90	36.60	40.90
Occupational position, %				
Manual	50.60	37.10^ab^	63.00	54.20
Nonmanual	36.50	47.70	26.00	33.10
Self-employed	12.90	15.20	11.00	12.70
Current smoking status, %	20.80	31.40^b^	16.20	21.40

Data were shown as median (interquartile range) or percentages (%). WC: waist circumference; MetS z-score (4 factors) includes BG, TG, HDL, and BP z-scores; abdominal WC: WC ≥ 90 cm for males and WC ≥ 80 cm for females; normal WC: <90 cm for males and <80 cm for females. Data were analyzed by adjusting for age and sex. ^a^ *P* value of <0.05 normal WC with NAFLD (2) compared with normal WC without NAFLD (1). ^b^ *P* value of <0.05 normal WC with NAFLD (2) compared with abnormal WC without NAFLD (3).

**Table 2:** Prevalence of MetS components and the odds ratios (ORs) for developing an abnormality among the study population, grouped by WC and NAFLD.

		Normal WC				Abnormal WC			
		Without NAFLD (1)		With NAFLD (2)		Without NAFLD (3)		With NAFLD (4)	
		*n* = 5159		*n* = 941		*n* = 1864		*n* = 1730	
		Per (%)	OR	Per (%)	OR (95% CI)	Per (%)	OR (95% CI)	Per (%)	OR (95% CI)
BG disorder	Crude	19.20	1.0	52.00	1.51 (1.30, 1.74)	34.40	1.67 (1.49, 1.86)	59.50	2.61 (2.33, 2.92)
	Adjusted		1.0		1.76 (1.50, 2.07)		1.22 (1.08, 1.37)		2.29 (2.03, 2.58)
TG disorder	Crude	15.40	1.0	20.20	4.57 (3.95, 5.28)	28.70	2.21 (1.97, 2.49)	34.20	6.20 (5.51, 6.98)
	Adjusted		1.0		3.96 (2.26, 6.12)		3.40 (1.98, 5.40)		4.62 (2.57, 6.94)
HDL disorder	Crude	46.40	1.0	59.90	1.39 (1.16, 1.65)	71.50	2.21 (1.95, 2.50)	78.70	2.84 (2.51, 3.22)
	Adjusted		1.0		2.06 (1.70, 2.50)		2.04 (1.77, 2.35)		2.99 (2.61, 3.44)
BP disorder	Crude	27.70	1.0	36.60	1.73 (1.50, 1.99)	38.90	2.89 (2.58, 3.25)	49.90	4.26 (3.75, 4.84)
	Adjusted		1.0		1.62 (1.38, 1.91)		2.05 (1.8, 2.34)		3.63 (3.15, 4.18)
≥2 MetS factors (exclude WC)	Crude	31.30	1.0	58.30	3.07 (2.66, 3.54)	55.90	2.78 (2.49, 3.10)	75.40	6.73 (5.94, 7.62)
	Adjusted		1.0		3.3 (2.82, 3.86)		2.13 (1.89, 2.40)		6.07 (5.32, 6.93)
≥3 MetS factors (exclude WC)	Crude	8.40	1.0	22.50	3.19 (2.66, 3.83)	23.20	3.32 (2.87, 3.84)	41.30	7.71 (6.72, 8.85)
	Adjusted		1.0		3.35 (2.76, 4.06)		2.67 (2.29, 3.12)		6.93 (6.00, 8.01)
4 MetS factors (exclude WC)	Crude	1.10	1.0	4.00	3.91 (2.57, 5.94)	5.40	5.32 (3.81, 7.42)	10.20	10.58 (7.77, 14.39)
	Adjusted		1.0		4.52 (2.91, 7.02)		3.57 (2.51, 5.06)		8.16 (5.93, 11.24)

Data were shown as percentages (%). Abdominal WC: WC ≥ 90 cm for males and WC ≥ 80 cm for females; normal WC: <90 cm for males and <80 cm for females. The estimates of the associations were adjusted for age, sex, living area, occupation, and smoking status.

**Table 3:** The values of insulin resistance and MetS z-score of 2, 3, or 4 disorder combinations of the study population, grouped by WC and NAFLD.

	Normal WC		Abnormal WC	
	Without NAFLD (1)	With NAFLD (2)	Without NAFLD (3)	With NAFLD (4)
Having 2 disorders (exclude WC)	*n* = 1186	*n* = 337	*n* = 609	*n* = 591
Insulin, pmol/L	30 (21.6, 41.7)	35.6 (25.6, 51.43)^a^	38.95 (27.8, 53.65)	46.9 (33.2, 64.83)
HOMA-IR	1.10 (0.81, 1.52)	1.35 (0.89, 1.81)^a^	1.39 (1.01, 1.99)	1.74 (1.19, 2.45)
MetS z-score (4 factors)	−0.82 (−1.80, 0.11)	−0.20 (−1.13, 0.74)^a^	−0.39 (−1.20, 0.63)	0.09 (−0.83, 1.23)
Having 3 disorders (exclude WC)	*n* = 376	*n* = 174	*n* = 332	*n* = 537
Insulin, pmol/L	33.3 (25.18, 46.15)	39.15 (27, 55.95)	42.2 (29.55, 57.6)	52.5 (37.7, 75.2)
HOMA-IR	1.27 (0.93, 1.82)	1.57 (1.10, 2.13)	1.62 (1.15, 2.41)	2.14 (1.51, 3.20)
MetS z-score (4 factors)	0.88 (−0.09, 2.07)	1.54 (0.40, 2.67)	1.46 (0.26, 2.86)	1.76 (0.69, 3.69)
Having 4 disorders (exclude WC)	*n* = 55	*n* = 38	*n* = 101	*n* = 177
Insulin, pmol/L	41.1 (27.85, 64)	52.15 (30.48, 63.5)	45.3 (32.65, 66.4)	60.1 (41.4, 83.75)
HOMA-IR	1.62 (1.26, 2.49)	2.15 (1.40, 3.10)	1.95 (1.33, 2.80)	2.73 (2.01, 4.10)
MetS z-score (4 factors)	2.74 (1.52, 5.93)	3.22 (1.56, 5.83)	2.68 (1.51, 4.17)	4.79 (2.77, 7.40)

Data were shown as median (interquartile range). Having *n* disorders means disorder combinations from abnormal BG, TG, HDL, and BP; MetS z-score (4 factors) includes BG, TG, HDL, and BP z-scores. Abdominal WC: WC ≥ 90 cm for males and WC ≥ 80 cm for females; normal WC: <90 cm for males and <80 cm for females. Data were analyzed by adjusting for age and sex. ^a^ *P* value of <0.05 normal WC with NAFLD (2) compared with normal WC without NAFLD (1). All parameters showed no differences between normal WC with NAFLD (2) and abnormal WC without NAFLD (3).

## Abbreviations

BMI: Body mass index
BP: Blood pressure
CVD: Cardiovascular disease
DM: Diabetes mellitus
FBG: Fasting blood glucose
HDL-C: High-density lipoprotein cholesterol
HOMA-IR: Homeostatic model assessment for insulin resistance
MetS: Metabolic syndrome
NAFLD: Nonalcoholic fatty liver disease
ORs: Odds ratios
TG: Triglyceride
VAT: Visceral adipose tissue
WC: Waist circumference.

## Supplementary Materials

Supplementary Materials Supplementary Table 1: metabolic profile and insulin resistance index of the study population (exclude subjects taking antidiabetic, antihypentensive, and antidyslipidemic agents, *n* = 1496).
