# Supplementary material for: Fatty Liver as a Potential Surrogate for Waist Circumference in the Diagnosis of Metabolic Syndrome: A Population-Based Study among Chinese Adults
Source: Int J Endocrinol. 2018 Apr 10;2018:7903982. doi: 10.1155/2018/7903982 (PMC5914088; doi:10.1155/2018/7903982)
Supplement: Supplementary Materials — Supplementary Table 1: metabolic profile and insulin resistance index of the study population (exclude subjects taking antidiabetic, antihypentensive, and antidyslipidemic agents, n = 1496). [file 7903982.f1.docx]

|  | Normal WC | | Abnormal WC | |
| --- | --- | --- | --- | --- |
|  | Without NAFLD | With NAFLD | Without NAFLD | With NAFLD |
|  | N=4705 | N=793 | N=1465 | N=1235 |
| TG, mmol/L | 1.08(0.82, 1.49) | 1.72(1.17, 2.44) ^ab^ | 1.35(0.99, 1.91) | 1.83(1.36, 2.58) |
| HDL, mmol/L | 1.47(1.28, 1.69) | 1.3(1.11, 1.49) ^a^ | 1.39(1.19, 1.6) | 1.29(1.13, 1.47) |
| SBP, mmHg | 123(111, 137) | 128(116, 142) ^ab^ | 134(122, 150) | 136(124, 151) |
| DBP, mmHg | 75(67, 84) | 80(72, 88) ^a^ | 80(72, 88) | 84(76, 92) |
| FPG, mmol/L | 5.18(4.84, 5.6) | 5.28(4.9, 5.82) ^a^ | 5.3(4.92, 5.8) | 5.46(5.03, 6.13) |
| Insulin, pmol/L | 28(20.18, 38.6) | 35.75(25.15, 50.5) ^a^ | 36.7(26.5, 50.3) | 48.1(35.5, 67.75) |
| HOMAIR | 0.94(0.66, 1.30) | 1.26(0.84, 1.75) ^a^ | 1.27(0.89, 1.77) | 1.74(1.23, 2.57) |
| MetS z-score  (4 factors) | -2.58(-3.76, -1.27) | -0.99(-2.36, 0.49) ^ab^ | -1.16(-2.51, 0.28) | 0.07(-1.31, 1.70) |

Supplementary Table 1: Metabolic profile and insulin resistance index of the study population (exclude subjects taking anti-diabetic, anti-hypentensive, anti-dyslipidemic agents, n=1496)

Data were analyzed by adjusting for age and sex.

a, P value of < 0.05 Normal WC With NAFLD (2) compared with Normal WC Without NAFLD (1).

b, P value of < 0.05 Normal WC With NAFLD (2) compared with Abnormal WC Without NAFLD (3).
